# Precise Measurement of Tellurium Isotope Ratios in Terrestrial Standards Using a Multiple Collector Inductively Coupled Plasma Mass Spectrometry

**DOI:** 10.3390/molecules25081956

**Published:** 2020-04-23

**Authors:** Rajamanickam Murugan, Tatsuo Aono, Sarata Kumar Sahoo

**Affiliations:** Environmental Radionuclide Research Group, National Institutes for Quantum and Radiological Science and Technology (QST), Chiba 263-8555, Japan; murugan.rajamanickam@qst.go.jp (R.M.); aono.tatsuo@qst.go.jp (T.A.)

**Keywords:** MC–ICP–MS, tellurium isotope ratios, terrestrial standards

## Abstract

Precise tellurium (Te) isotope ratio measurement using mass spectrometry is a challenging task for many decades. In this paper, Te isotope ratio measurements using multi-collector inductively coupled plasma mass spectrometry (MC–ICP–MS) in terrestrial Te standards have been reported. Newly developed Faraday cup with 10^12^ Ω resistor is used to measure low abundance ^120^Te, whereas the 10^11^ Ω resistor is used to measure other Te isotopes. The relative standard deviation obtained for Te isotope ratio measurement by Faraday cups of ^120^Te/^128^Te [0.002907(05)], ^122^Te/^128^Te [0.079646(10)], ^123^Te/^128^Te [0.027850(07)], ^125^Te/^128^Te [0.221988(09)], ^126^Te/^128^Te [0.592202(20)], and ^130^Te/^128^Te [1.076277(30)] were 0.140%, 0.014%, 0.026%, 0.005%, 0.004%, and 0.004%, respectively. The measured isotope ratio results are compared with previous results obtained by thermal ionization mass spectrometry (TIMS), negative thermal ionization mass spectrometry (N–TIMS), and MC–ICP–MS, showing an improvement in the precision about one order of magnitude for ^120^Te/^128^Te ratio. The present study shows better precision for Te isotope ratios compared to earlier studies.

## 1. Introduction

The origin of elements suggests that a number of different nucleosynthesis processes produce the present-day abundances of the elements. It is assumed that the products of the nucleosynthetic processes have been injected into our solar system from stars and supernovae, a mixture of debris together with intergalactic hydrogen and helium. The basic processes such as rapid (r), slow (s) neutron-capture processes, and proton (p) capture processes are involved in heavy-element synthesis. The r- and p-process elements are formed in different environments compared to the s-process. Therefore, earlier researchers proposed the possibility of inhomogeneity or mixing may have occurred between the process, during, or after the period of supernova injection. The isotopic heterogeneity in terrestrial and meteoritic samples are either produced by isotopic fractionation or by incomplete mixing of material with different nucleosynthesis histories during the formation of the solar system [1]. Cameron [2] suggested that accurate isotopic composition measurement on heavy elements with even atomic numbers reveal the anomalies between p-, r-, and s-process nuclides. The elements which have a comparable abundance from p- and s-process are ideal for checking Cameron’s hypothesis. Tellurium (Te) is an ideal element to check the hypothesis. It has eight naturally occurring isotopes with a mass number 120 (0.10% nominal natural abundances), 122 (2.60%), 123 (0.91%), 124 (4.82%), 125 (7.14%), 126 (19.0%), 128 (31.7%), and 130 (33.8%), exhibiting a large number of oxidation states (−2, 0, +4, +6). Te is unique; it is the only element that possesses one p-process isotope of ^120^Te, three s-process isotopes of ^122^Te, ^123^Te, ^124^Te, and r-process isotopes of ^128^Te, ^130^Te. The ^125^Te, ^126^Te isotopes are produced by both s- and r-processes. The ^126^Te is formed by the beta decay of ^126^Sn with a half-life of 234,500 years. This decay system is also used as a possible chronometer to understand the processes which occurred during the early solar system [3,4]. 

Analysis of Te isotopic composition is primarily difficult due to its high first ionization potential (~9 eV), which decreases analytical sensitivity. Several researchers have attempted various methods to measure Te isotopic composition for the past five decades. Initially, Te isotope ratios were measured using thermal ionization mass spectrometry (TIMS) with Re filaments [5,6,7]. The low ionization efficiency, relatively low natural abundances, and high sample amount restricted the development of Te isotope measurement using TIMS. In 1992, Wachsmann and Heumann [8] investigated the formation of negative ions for Se and Te isotopes, as higher electron affinity will produce high negative thermal ions. Thus, Te isotopic composition measurement using negative TIMS with an Re double filament and Ba(OH)_2_ activator achieved a better precision by a factor of 10 [8]. In 2014, Fukami and Yokoyama measured Te isotope ratios using N–TIMS, achieving a good sensitivity and uncertainties of less than 0.01%. However, N–TIMS requires a significantly higher concentration of Te than that is required for ICP–MS measurements [9,10]. Subsequently, multi-collector inductively coupled plasma mass spectrometry (MC–ICP–MS) paired with a desolvating nebulizer is used to achieve high improvement in accuracy and precision for the Te isotope ratio measurement, due to the ionization of a greater portion of Te atoms [11,12,13,14,15,16,17]. 

MC–ICP–MS is a good option to improve the precision of the isotope ratio measurement. The key drawback of MC–ICP–MS is the use of relatively low sensitivity Faraday cup detectors compared to the ion counting systems. Some of the MC–ICP–MS are equipped with a multiple ion counting system. Due to the high intensity of ions, they are easily damaged by false operations. To achieve the high sensitivity, Faraday cups equipped with 10^12^ Ω resistors in the feedback loop are used [18,19,20]. The new Faraday cup with a highly resistive 10^12^ Ω resistor can enhance the signal intensity and background noise by approximately 10 and three times, respectively. It is very useful for determining a small number of samples or signals [21,22]. The application of the new 10^12^ Ω resistor has enabled both high sensitivity and precision. The hydride generation (HG) technique with MC–ICP–MS for the Te isotopic measurement, significantly improves ionization efficiency, analytical sensitivity, and limits potential isobaric interferences [23,24].

In this contribution, the analytical performance of MC–ICP–MS with a desolvating nebulizer sample introduction system employing the new Faraday cup of 10^12^ Ω resistors are used for the determination of low abundance ^120^Te. To demonstrate the applicability of this method Te isotope ratios were measured on terrestrial Te standards from different parts of the world, to detect any isotope heterogeneity or incomplete mixing present among the terrestrial standards.

## 2. Results and Discussion

### 2.1. Isotope Ratios of Te in a Reagent Standard

The Kanto chemical Te standard solution was used to evaluate the instrument protocol and demonstrate significant improvements in the exemption of isobaric interferences during MC–ICP–MS measurement. Te isotope ratios of Te standard measured on seven different dates from October 2019 to January 2020 are summarized in Table 1 and analytical uncertainties are expressed in two mean standard deviations (2σ_m_). 

**Figure 1:**
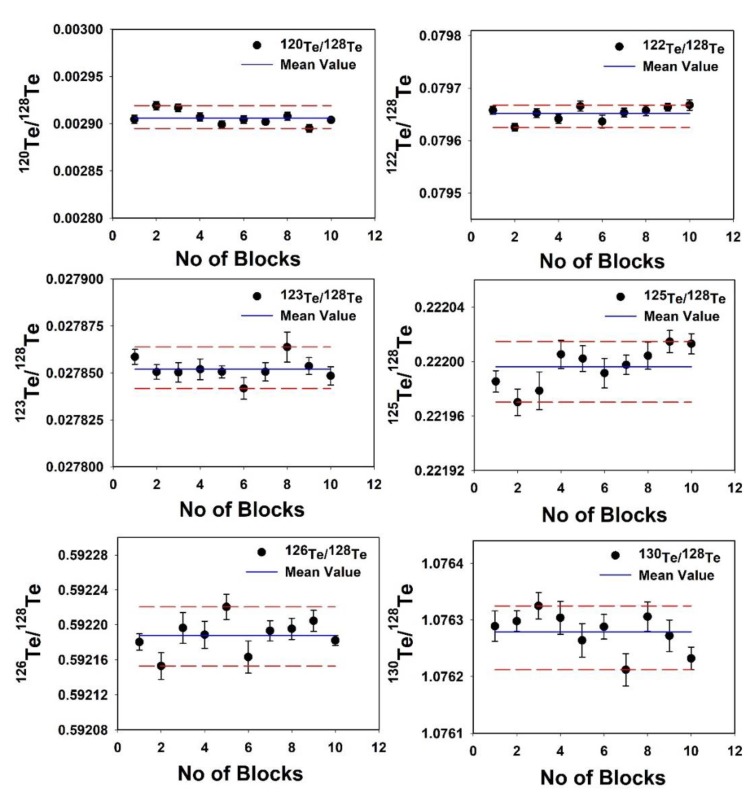
Stability of the isotope ratio measured using multi-collector inductively coupled plasma mass spectrometry (MC–ICP–MS). The dotted red line represents two standard errors (2SE) of a single measurement. Each block represents the mean of 20 individual ratios.

**Figure 2:**
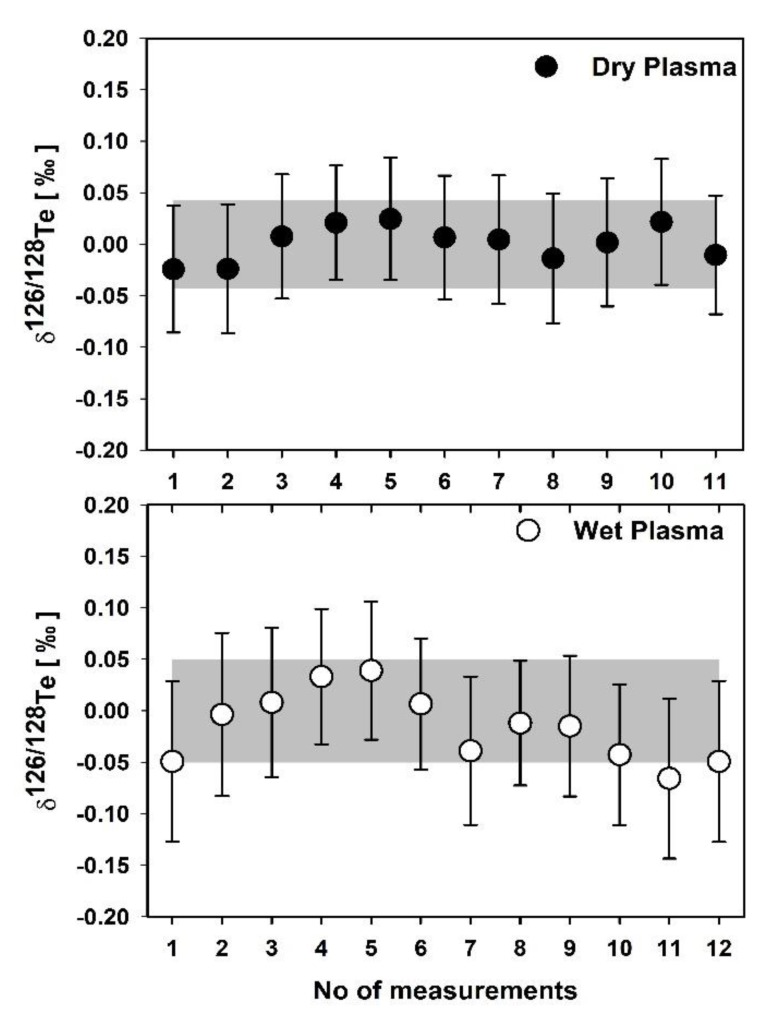
Reproducibility of Te isotope ratios analyses for the Kanto standard in wet and dry plasma conditions. The error bars show two standard deviations (2SD) from 10 block measurements in one run, representing the external reproducibility of the wet and dry plasma modes of the standard. Shaded zones depict 2SD of all standard runs for wet and dry plasma, respectively. Measurements were performed from October 2019 to January 2020 (seven analytical sessions for each plasma mode).

The stability of Te isotope ratio measurements is checked by one single measurement, which consists of 10 blocks, each block representing the mean of 20 individual ratios (Figure 1). The typical instrument sensitivity was 50 and 700 V per µg·mL^−1^ (total Te) under wet and dry plasma modes, respectively. The background signals were higher for dry plasma compared to the wet plasma. We evaluated the external reproducibility (two standard deviations) of the δ^126/128^Te measurement using laboratory Kanto Te standard in wet and dry plasma modes and are found as 0.034‰ and 0.018‰, respectively (Figure 2). Similarly, four other terrestrial Te standards were measured in both plasma modes. It shows the reproducibility of average δ^126/128^Te was 0.03 ± 0.02 and 0.01 ± 0.02 for wet and dry plasma modes, respectively. Thus, illustrating that dry plasma mode of Te measurement gives better reproducibility compared to the wet plasma mode. 

**Figure 3:**
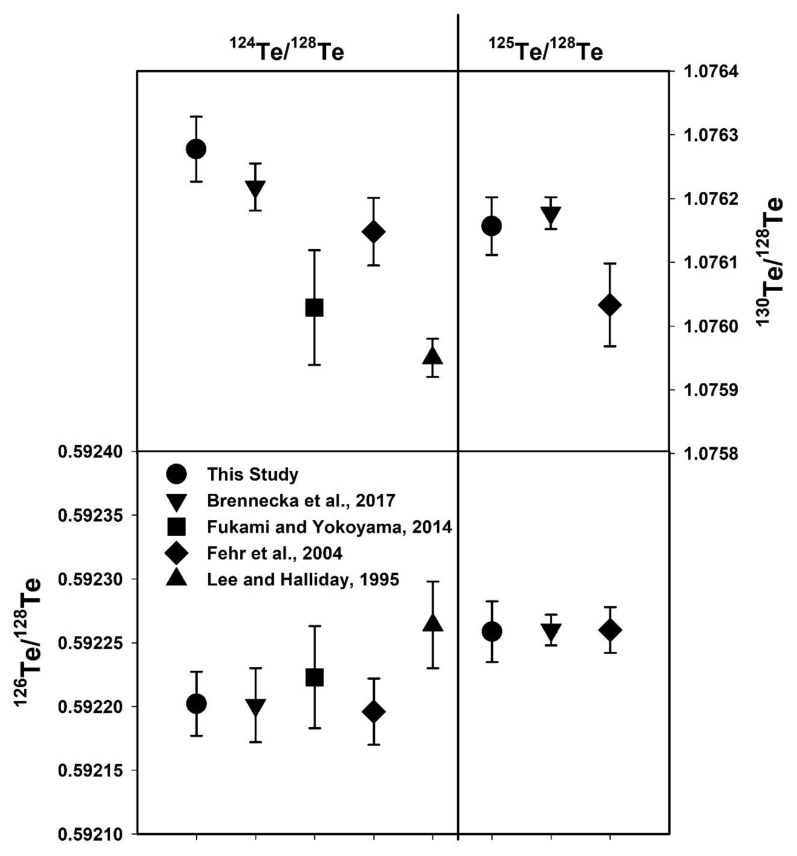
Data comparison between this study and recent studies of the Te isotopic composition for ^126^Te/^128^Te and ^130^Te/^128^Te ratios. The two different internal normalizations are shown on the top for comparison.

**Figure 4:**
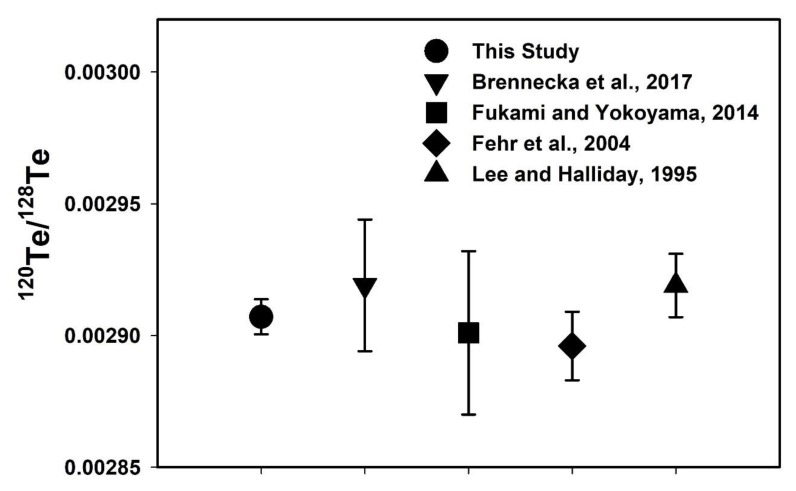
Data comparison between this study and recent studies of the Te isotope ratio of ^120^Te/^128^Te ratio normalization with ^124^Te/^128^Te ratio.

In this study, ^126^Te/^128^Te and ^130^Te/^128^Te ratios were measured using two different normalizations and are shown in Figure 3. The Te isotope ratios measured here are in good agreement with previously measured Te isotope ratios using different mass spectrometry instruments [5,6,8,10,11,12,23] (Table 3). However, there is a slight disagreement among data sets reported for ^130^Te/^128^Te ratios [17,23]. The measured ^130^Te/^128^Te isotope ratio was 1.076277 ± 57 and a little higher than previous studies (Table 3), but they are within the analytical error (95% confidence interval) except for the data of Fukami and Yokoyama [10] and Lee and Halliday [11] (Figure 3). The higher ^130^Te/^128^Te isotope ratio was earlier reported by correction of Xe interference with Te isotopes (^124^Te, ^126^Te, ^128^Te, ^130^Te) during measurement [12,23]. It was noticed that the ^126^Te/^128^Te ratio was in good agreement with earlier studies, especially compared with similar normalizations (Figure 3). Especially, the introduction of a 10^12^ Ω resistor for measuring ^120^Te ions reproduced better results of one order of magnitude for the ^120^Te/^128^Te isotope ratio compared to the previous studies (Figure 4). The measured ^120^Te/^128^Te and other Te isotope ratios in this study are within the analytical error range as shown in Table 3 (Brennecka et al. [23], Fukami and Yokoyama [10], Fehr et al. [12], Lee and Halliday [11], De Laeter [6], Wachsmann and Heumann [8], and Smith and De Laeter [5]). However, Loss et al. [7] reported all Te isotope ratios consistently lower values except the ^130^Te/^128^Te isotope ratio which could be due to the mass discrimination correction factor.

### 2.2. Stable Isotope Variation of Different Terrestrial Standards

The reproducibility is expressed in terms of delta (δ) notation. The δ^X/128^Te values were calculated based on Equation (1) relative to the mean of Te isotope ratios of laboratory Te standard measured on seven different dates. X stands for Te isotopes (120, 122, 123, 125, 126, 130).

(1) $$\delta^{X/128}\mathrm{Te}\;=\frac{\left(R_{\mathrm{Sample}}-R_{\mathrm{Standard}}\right)}{R_{\mathrm{standard}}}\times 10^{3}$$

The standard value, R _standard_, corresponds to the mean of laboratory standard Te isotope ratios, where R _Sample_ is ^120^Te/^128^Te, ^122^Te/^128^Te, ^123^Te/^128^Te, ^125^Te/^128^Te, ^126^Te/^128^Te, and ^130^Te/^128^Te ratio individually measured in terrestrial Te standard. 

Using the above-explained method, we measured Te isotope ratios of four terrestrial atomic absorption standards, Wako Chemical, Aldrich Chemical, Johnson Matthey and Spex CertiPerp. The Te isotope ratios of four terrestrial Te standards were measured six times on different dates from October 2019 to January 2020 and the mean values were compiled and given in Table 2. 

**Figure 5:**
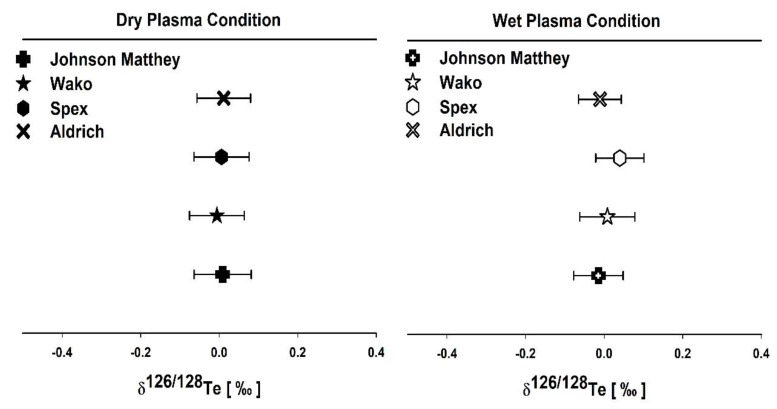
The dry and wet plasma measurements of δ^126/128^Te for four terrestrial Te standards plotted with respect to the Kanto standard (Kanto Chemical). The error bars are expressed in 2SD.

The analytical uncertainties are expressed in two standard deviations (2SD) to check the credibility of our measurements. Figure 5 shows the reproducibility of Wako Chemical, Aldrich Chemical, Johnson Matthey, and Spex CertiPerp which are in the range from –0.01 ± 0.06 to 0.04 ± 0.06 for δ^126/128^Te. 

There was no isotopic variation observed among the terrestrial Te standards with respect to laboratory Te standard for δ^126/128^Te. This implies that the atomic adsorption standard solutions measured in this current study from different parts of the world show similar isotope compositions and they do not show any anomaly for δ^126/128^Te. It implicates that the isotopes of Te are homogeneous in nature.

### 2.3. Precision and Reproducibility

The long-term reproducibility was evaluated from the data measured over a period of six months from October 2019 to January 2020. The reproducibility (2SD) observed for the Te measurement are 3.24‰, 0.13‰, 0.74‰, 0.02‰, 0.01‰ and 0.02‰ for δ^120^Te/^128^Te, δ ^122^Te/^128^Te, δ ^123^Te/^128^Te, δ ^125^Te/^128^Te, δ ^126^Te/^128^Te, and δ^130^Te/^128^Te for dry plasma mode, respectively. The significant difference between the measured Te isotope ratio among the Te standards with respect to laboratory standard results is checked, taking into account the measured results and their uncertainties. In this process, the absolute difference (AD) of the measured values are compared to the uncertainty of the absolute difference (U_AD_) [25]. The AD and U_AD_ are calculated by the following equation (Equations (2) and (3)).

(2) AD=|R−M|

(3) $$U_{\mathrm{AD}}\;=\;\sqrt{{\left(U_{R}\right)}^{2}+{\left(U_{M}\right)}^{2}}$$

where R and M are the Te isotope ratio of terrestrial Te standards (Johnson Matthey, Wako, Spex, Aldrich) and laboratory Te standard, respectively. U_R_ and U_M_ are the uncertainty of terrestrial Te standards (Johnson Matthey, Wako, Spex, Aldrich) and laboratory Te standard, respectively. If AD ≤ 1.96 U_AD_, then the isotope ratios measured in laboratory Te standard and terrestrial Te standards from this study are accepted as equal, corresponding to a confidence interval of 95%. All the Te isotope ratios of Te standards show AD values less than 1.96 U_AD_ and are within the error range. No larger isotopic variations were observed among the terrestrial Te standards isotope ratios with respect to the laboratory Te standard. The external precision of ^120^Te/^128^Te, ^122^Te/^128^Te, ^123^Te/^128^Te, ^125^Te/^128^Te, ^126^Te/^128^Te, and ^130^Te/^128^Te isotope ratios were 0.140%, 0.014%, 0.026%, 0.005%, 0.004%, and 0.004% relative standard deviations, respectively. The internal precisions obtained for the laboratory Te standard for ^120^Te/^128^Te, ^122^Te/^128^Te, ^123^Te/^128^Te, ^125^Te/^128^Te, ^126^Te/^128^Te, and ^130^Te/^128^Te are 114 ppm, 9 ppm, 16 ppm, 4 ppm, 3 ppm, and 3 ppm, respectively. The internal and external precisions are almost similar. 

### 2.4. Comparison with Previous Studies

Previously, many researchers have used terrestrial Te standards as an in-house standard for their studies using different mass spectrometric techniques. Therefore, we compared our present Te isotope ratio results with previously measured results to detect any variations among the isotope ratios measured through time. The data sets are compiled and given along with our present result in Table 3. The reproducibility of δ^120/128^Te, δ^123/128^Te, δ^125/128^Te, δ ^126/128^Te, and δ^130/128^Te of the present study was compared with the previous studies for comparison. 

**Figure 6:**
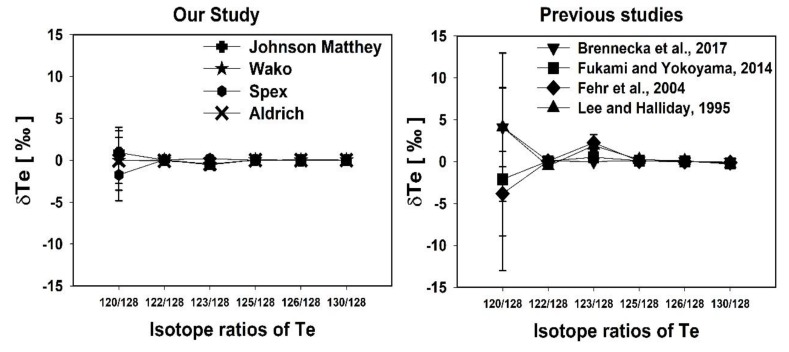
Results obtained for δ^120/128^Te, δ^123/128^Te, δ^125/128^Te, δ^126/128^Te, and δ^130/128^Te of this study was compared with the previous studies in-house Te standards. The error bars (2SD) denotes the external reproducibility of the measurement.

The δ^120^/^128^Te plot in a range of −3.82–4.09‰, δ^123/128^Te in range of −0.55–2.25‰, δ^126/128^Te in range of −0.01–0.10‰, and δ^130/128^Te in range of −0.30–0.02‰ (Figure 6). The δ^126/128^Te matches with previous studies and δ^130/128^Te shows a slight deviation compared to previous studies, but the deviation is less and within the analytical error. δ^120/128^Te and δ^123/128^Te show a relatively smaller deviation compared to the previous studies. The ^120^Te and ^123^Te have a direct interference with Sn and Sb. Therefore, using a special resistor (10^12^ Ω) for measuring ^120^Te and ^118^Sn and correcting for those interfering isotopes on ^120^Te and ^123^Te leads to the achievement of precise isotope ratio results in this study. The other reason could be either due to their very low natural abundance or because of two different nucleosynthesis processes such as p-process and s-process. However, the deviation of δ^120/128^Te and δ^123/128^Te among standard solutions is still unclear.

## 3. Material and Methods

### 3.1. Standards and Reagents

Five commercially available terrestrial Te standards have been used for the measurement of Te isotope ratios. Atomic absorption spectrometry standard solutions were obtained from Kanto Chemical (Lot no.103G9089, Kanto Chemical Co. Inc., Tokyo, Japan), Wako Chemical (Lot. No. JCF9875 Wako pure chemical industries, Ltd., Osaka, Japan), Aldrich Chemical (Lot no.12329LR, Aldrich Chemical Company, Inc., Milwaukee, WI, USA), Spex CertiPerp (Lot No. 6-250TE, Spex CertiPerp Company, Inc., Metuchen, NJ, USA), and Johnson Matthey Chemicals (Lot No. 801141G, Royston, UK). All standard solutions were concentration at 1000 µg·mL^−1^. The dilution was carried out in a class 100 laminar flow hood using deionized water (>18 MΩ·cm^−1^) produced with a Milli-Q system (Merck Millipore, Burlington, MA, USA) and Tamapure AA-100 ultrapure (Tama Chemicals, Kawasaki, Japan) HNO_3_. 

### 3.2. Instrumentation 

Te isotope ratio measurements were performed using a Nu Plasma 3D (Nu Instruments Ltd., Wrexham, UK) MC–ICP–MS at National Institute for Quantum and Radiological Sciences (QST), in low-resolution mode using nickel sampler cone and nickel wide-angle skimmer cone. MC–ICP–MS consisted of fixed 21 collectors; among these, 16 were Faraday cups and 5 were Daly collectors (Table 5). The L6 and L7 Faraday cups were equipped with a fixed 10^12^ Ω resistor. Ax, H2, H3, H4, H5, H6, H7, and H8 Faraday cups were equipped with fixed 10^11^ Ω resistors. H1, L1, L2, L3, L4, and L5 Faraday cups were equipped with a switchable dual resistors setup of 10^11^ Ω and 10^12^ Ω, respectively. The switching of the resistor for Faraday cups was performed using the Nu Plasma software. Gain calibration of each Faraday cup resistors was performed every day using the software operated standard procedure by supplying 4 V. It is consecutively applied for all the Faraday cups with 10^11^ Ω to 10^12^ Ω resistors. The gain values were within 20 ppm error range.

Recently, researchers have used Faraday detection systems with amplifiers equipped with 10^12^ Ω and 10^13^ Ω resistors in the feedback loop for the measurements of low ion intensities [19,20]. The 10^12^ Ω resistor provides 10 times higher voltage compared to 10^11^ Ω resistor for a given ion beam, whereas the Johnson–Nyquist (JN) noise level of the resistor increases by a factor of $\sqrt{10}$. Therefore theoretical 3-fold improvement in the signal to noise ratio is expected but, in practice, this ratio improves only by a factor of two. 

The response time of the 10^12^ Ω resistors takes time for the signal on the resistors to reach their baseline value, which is slower compared to the 10^11^ Ω resistors. The curve fitting decay time for each resistor was determined by measuring the signal after closing the second line of sight valve using the Nu Plasma software. The tau corrections were carried out for the 10^12^ Ω resistor. Therefore, a relatively slow response does not affect the data quality of MC–ICP–MS analytical performance. 

### 3.3. Te Isotope Ratio Measurement Protocol of MC–ICP–MS.

The operating conditions of MC–ICP–MS for Te isotope ratio measurements are given in Table 4. Prior to each measurement session, the instrument was carefully tuned to maximum Te signal intensity by adjusting the torch position, Ar gas flow, lens voltages, and deflector settings. ^126^Te isotope mass is selected as monitoring isotope for peak centering before the measurement of each block. On-peak background subtraction was performed using beam intensities that were measured by introducing 2% HNO_3_ before sample measurement.

Kanto chemical Te standard was used as laboratory standard and Te isotope ratio measurements were carried out in both wet plasma and dry plasma mode. In wet plasma mode, the measurement was performed using a 200 ng·mL^−1^ Te concentration solution. Sample solutions were introduced into the plasma through a micromist nebulizer with an aspiration rate of 200 µL·min^−1^. After each measurement, a washout was performed with 2% HNO_3_ for 15 min. The isotope ratio measurement comprises 10 blocks of 20 cycles with an 8 s integration time. One block consists of 20 cycles (isotope ratios). 

In dry plasma mode, Aridus-3 desolvating nebulizer (Teledyne CETAC Technologies, USA) was used where 10 ng·mL^−1^ Te concentration solution was aspirated at a rate of 100 µL·min^−1^. The Ar sweep gas flow rate was typically 4.25 L·min^−1^ with a nebulizer gas flow rate of 0.9 L·min^−1^. No additional N_2_ gas was used for the measurement. The other instrumental operating settings are identical to wet plasma measurements. The washout was approximately 30 min using a 2% HNO_3_. Te standard with a concentration of 10 ng·mL^−1^ and 200 ng·mL^−1^ was analyzed before and after every terrestrial Te standard to confirm the absence of drift in dry and wet plasma mode of measurement, respectively. The amount of Te consumed in one measurement was approximately 27 ng and 1067 ng for dry and wet plasma mode, respectively.

There were 11 Faraday cups used for the simultaneous collection of ion beams. The L5 Faraday cup resistor setup was changed from 10^11^ Ω to 10^12^ Ω resistor. The measurement of Te isotope ratios was carried out using Faraday cups with a mixed resistor of 10^11^ Ω and 10^12^ Ω in a static cup configuration mode. The ^118^Sn, ^120^Te, were collected using L6 and L5 10^12^ Ω resistor Faraday cups, and ^121^Sb, ^122^Te, ^123^Te, ^124^Te, ^125^Te, ^126^Te, ^128^Te, ^129^Xe, and ^130^Te were detected using L4, L3, L2, L1, Ax, H1, H3, H4, and H5, 10^11^ Ω resistor Faraday cups, respectively (Table 5). 

The Te isotope ratios (all ratios) obtained from the Faraday cups were corrected for mass fractionation by normalizing with ^124^Te/^128^Te = 0.14853 [11] and ^125^Te/^128^Te = 0.22204 [1] using exponential fractional law. However, the previously published isotope results have used different normalization ratios to correct for internal normalization. ^124^Te/^128^Te normalization was carried out for TIMS and N–TIMS measurements. Due to ionization potential, isobaric interference of Sn and Xe could be controlled [6,10]. The direct comparison of data with the previously published data is possible [10,11,12,15,23]. Since there is no isobaric interference of Xe or Sn with ^125^Te, it was preferred in MC–ICP–MS studies. The internal normalization of ^125^Te/^128^Te was selected for mass bias correction, because the mass-bias-corrected ^126^Te/^128^Te and ^130^Te/^128^Te isotope data do not require any Sn correction, which is very much important for the sample containing a significant amount of Sn.

The possible isotopic interference for Te isotopes was listed and the interference corrections for Te isotopes were carried out by the earlier workers [11,12,23]. A similar correction procedure was followed for the Te isobaric correction in this study. The major isobaric interferences on Te isotopes can be generated from Sn, Sb, and Xe. Therefore, the ion currents of ^118^Sn^+^, ^121^Sb^+^, and ^129^Xe^+^ were measured during the measurement to do interference corrections. Xenon is present in the Ar plasma gas and the typically reported yield of Xe/Te ratios are 4–9 × 10^−4^ [12]. The correction of ^118^Sn on ^120^Te, ^122^Te, ^121^Sb on ^123^Te, ^124^Te, and ^129^Xe on ^124^Te, ^126^Te, ^128^Te, and ^130^Te isotopes were applied online during the measurement using exponential law. The intensities of ^118^Sn, ^121^Sb, and ^129^Xe were 2.3 × 10^−16^ A, 2.05 × 10^−15^ A, and 6.28 × 10^−16^ A for wet plasma condition, and 1.5 × 10^−16^ A, 8.8 × 10^−16^ A, and 9.6 × 10^−15^ A for dry plasma, respectively. The typical production rate of tellurium hydride (TeH) was 2 × 10^−4^ and <1 × 10^−5^ for wet and dry plasma, respectively. The TeH correction was not necessary for the dry plasma condition because of its low production rate [15]. As it is already mentioned by the earlier studies that Te standard solutions typically displayed Sn/Te concentration ratios of about 1–3 × 10^−4^ and Sn/Te ratios of up to 1.5 × 10^−3^ could be tolerated for samples without compromising the accuracy of the analytical results [12]. 

## 4. Conclusions

Analytical performance of MC–ICP–MS method employing a Faraday cup with a new 10^12^ Ω resistor was examined. Use of a special resistor (10^12^ Ω) Faraday cup for measuring ^120^Te shows 10 times detection efficiency and improves the precision by one order of magnitude for ^120^Te/^128^Te isotope ratio compared to previous studies. The reproducibility (2SD) observed for the Te isotope ratio measurement using dry plasma conditions are 3.24‰, 0.13‰, 0.74‰, 0.02‰, 0.01‰, and 0.02‰ for δ^120/128^Te, δ^122/128^Te, δ^123/128^Te, δ^125/128^Te, δ^126/128^Te, and δ^130/128^Te, respectively. Furthermore, the Te terrestrial atomic adsorption standards used in this study from different parts of the world are isotopically homogeneous and do not show any incomplete mixing in nature. In the future, the environmental and meteorite samples can be measured precisely using this protocol of MC–ICP–MS, which shows better precision compared to the previous studies.

## Figures and Tables

**Table 1:** Te isotope ratios of Kanto Chemical Reagent Standard.

S. No	^120^Te/^128^Te	^122^Te/^128^Te	^123^Te/^128^Te	^125^Te/^128^Te	^126^Te/^128^Te	^130^Te/^128^Te
1	0.002906 (07)	0.079652 (10)	0.027852 (06)	0.221996 (10)	0.592188 (20)	1.076257 (40)
2	0.002900 (03)	0.079650 (10)	0.027854 (06)	0.221991 (10)	0.592188 (10)	1.076303 (30)
3	0.002908 (04)	0.079629 (10)	0.027857 (09)	0.221991 (08)	0.592207 (10)	1.076290 (30)
4	0.002902 (02)	0.079654 (10)	0.027845 (06)	0.221990 (06)	0.592214 (20)	1.076222 (30)
5	0.002915 (06)	0.079636 (06)	0.027861 (09)	0.221981 (10)	0.592217 (20)	1.076279 (30)
6	0.002889 (06)	0.079642 (08)	0.027861 (08)	0.221966 (07)	0.592206 (20)	1.076307 (20)
7	0.002928 (03)	0.079656 (08)	0.027822 (04)	0.222002 (08)	0.592196 (10)	1.076284 (30)
	**0.002907 (05)**	**0.079646 (10)**	**0.027850 (07)**	**0.221988 (09)**	**0.592202 (20)**	**1.076277 (30)**

Uncertainties are expressed in two sigma mean (2σ_m_). All the isotopes are normalized to ^124^Te/^128^Te = 0.14853 using the exponential law.

**Table 2:** Tellurium isotope data of terrestrial standards.

S. No	Standards	δ^120/128^Te	δ^122/128^Te	δ^123/128^Te	δ^125/128^Te	δ^126/128^Te	δ^130/128^Te
1	Aldrich	−0.03 ± 2.76	−0.07 ± 0.25	−0.45 ± 0.39	0.004 ± 0.10	−0.01 ± 0.05	0.01 ± 0.07
2	Spex	−1.75 ± 3.11	0.05 ± 0.28	0.18 ± 0.44	−0.01 ± 0.11	0.04 ± 0.06	0.02 ± 0.08
3	Wako	−0.03 ± 3.56	0.03 ± 0.32	−0.55 ± 0.50	0.03 ± 0.13	0.01 ± 0.07	0.02 ± 0.09
4	Johnson Matthey	0.90 ± 3.05	0.02 ± 0.28	−0.46 ± 0.44	0.03 ± 0.11	−0.01 ± 0.06	0.01 ± 0.07

Uncertainties are expressed in 2SD. All the isotopes are normalized to ^124^Te/^128^Te = 0.14853 using the exponential law.

**Table 3:** Comparison of Tellurium isotope data acquired from this study with previously published results.

^125^Te/^128^Te Normalization	**Method**	**Standards**	**^120^** **Te/^128^Te**	**^122^** **Te/^128^Te**	**^123^** **Te/^128^Te**	**^124^** **Te/^128^Te**	**^125^** **Te/^128^Te**	**^126^** **Te/^128^Te**	**^130^** **Te/^128^Te**
This Study (*n* = 7)	**MC–ICP–MS**	**Kanto**	**0.002903 ± 06**	**0.079665 ± 14**	**0.027852 ± 07**	**0.148559 ± 13**	**0.222040**	**0.592259 ± 27**	**1.076152 ± 45**
Brennecka et al. (2017)	HG–MC–ICP–MS	Spex	0.002916 ± 13	0.079666 ± 06		0.148550 ± 09	0.222040	0.592260 ± 12	1.076177 ± 25
Fehr et al. (2004)	MC–ICP–MS	Alfa Aesar	0.002897 ± 13	0.079678 ± 11	0.027921 ± 26	0.148563 ± 15	0.222040	0.592260 ± 18	1.076033 ± 65
^124^Te/^128^Te Normalization									
This Study (*n* = 7)	**MC–ICP–MS**	**Kanto**	**0.002907 ± 07**	**0.079646 ± 16**	**0.027850 ± 09**	**0.14853**	**0.221988 ± 16**	**0.592202 ± 25**	**1.076277 ± 57**
Brennecka et al. (2017)	HG–MC–ICP–MS	Spex	0.002919 ± 25	0.079651 ± 9		0.14853	0.222014 ± 13	0.592201 ± 29	1.076218 ± 37
Fukami and Yokoyama (2014)	N–TIMS	Kanto	0.002901 ± 31	0.079650 ± 34	0.027865 ± 12	0.14853	0.222011 ± 29	0.592223 ± 40	1.076029 ± 90
Fehr et al. (2004)	MC–ICP–MS	Alfa Aesar	0.002896 ± 13	0.079650 ± 11	0.027913 ± 26	0.14853	0.222003 ± 16	0.592196 ± 26	1.076148 ± 53
Lee and Halliday (1995)	MC–ICP–MS	Johnson Matthey	0.002919 ± 12	0.079603 ± 16	0.027904 ± 12	0.14853	0.222041 ± 25	0.592264 ± 34	1.075950 ± 30
De Laeter (1994)	TIMS	Johnson Matthey	0.002891 ± 32	0.079492 ± 44	0.027878 ± 33	0.14853	0.221722 ± 61	0.59153 ± 18	1.07889 ± 14
Wachsmann and Heumann (1992)	N–TIMS	Johnson Matthey		0.07987 ± 44	0.02774 ± 21	0.14853	0.22199 ± 69	0.5919 ± 22	1.0752 ± 10
Loss et al. (1990)	TIMS		0.002731 ± 72	0.07716 ± 19	0.027404 ± 99	0.14853	0.211754 ± 57	0.57392 ± 10	1.110642 ± 84
Smith and De Laeter (1986)	TIMS	Johnson Matthey	0.002895 ± 31	0.07965 ± 26	0.027884 ± 64	0.14853	0.22200 ± 30	0.59235 ± 63	1.07571 ± 46

Uncertainties are expressed in 2SD (two standard deviations).

**Table 4:** MC–ICP–MS operating and measurement conditions.

**RF Power**		1300 W
**Acceleration Potential (V)**		6000
Sampler cone		Ni cone
Skimmer cone		Ni wide-angle cone
Resolution		Low
Cool gas		13.4 L·min^−1^
Auxiliary gas		0.90 L·min^−1^
	**Wet Plasma**	**Dry Plasma**
Sample	Conventional Spray chamber	Desolvating Nebulizer
Nebulizer	Micromist, 200 µL·min^−1^	C-Flow PFA, 100 µL·min^−1^
Nebulizer gas	1.14 L·min^−1^	0.90 L·min^−1^
Sweep Ar Gas	----	4.2 L·min^−1^
N_2_ gas	----	0 L·min^−1^
Sample Concentration	200 ng·mL^−1^	10 ng·mL^−1^
Typical Sensitivity	50 V per µg·mL^−^^1^	700 V per µg·mL^−1^
Washout time	10–15 min	30 min
^130^Te Beam intensity	1.60 V	1.46 V

**Table 5:** Configuration of collectors in MC–ICP–MS.

Detectors	L7	D4	D3	D2	D1	L6	D0	L5	L4	L3	L2	L1	Ax	H1	H2	H3	H4	H5	H6	H7	H8
Monitored Isotopes								^120^Te		^122^Te	^123^Te	^124^Te	^125^Te	^126^Te		^128^Te		^130^Te			
Isobaric Interference						^118^Sn			^121^Sb								^129^Xe				
Faraday Cup resistors	10^12^ Ω					10^12^ Ω		10^12^ Ω	10^11^ Ω	10^11^ Ω	10^11^ Ω	10^11^ Ω	10^11^ Ω	10^11^ Ω	10^11^ Ω	10^11^ Ω	10^11^ Ω	10^11^ Ω	10^11^ Ω	10^11^ Ω	10^11^ Ω

L refers to lower side Faraday cups, H refers to higher side Faraday cups with respect to the axial (Ax) Faraday cup, and D stands for Daly detectors.
